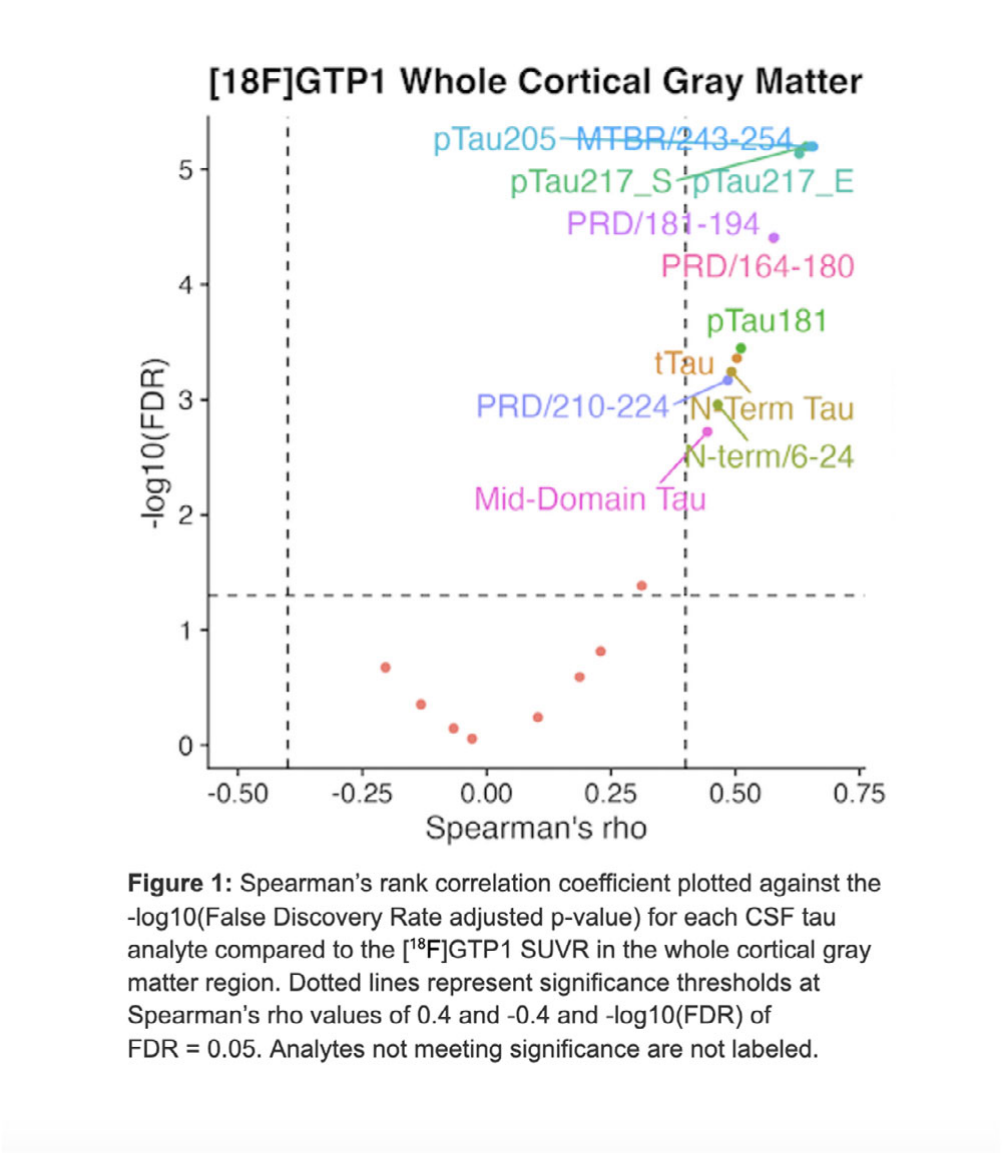# Identification of CSF and brain tau species in Alzheimer's Disease that are highly correlated with tau pathology

**DOI:** 10.1002/alz70856_101355

**Published:** 2025-12-25

**Authors:** Julie Lee, Balazs Toth, Sandra Sanabria Bohorquez, Stephen Schauer, Oded Foreman, Meena Choi, Tobias Bittner, Miriam Baca, Monika Dohse, Hai Ngu, Anne Biever, Emilia Calderon, Melanie Mandl, Norbert Wild, Kristina Bierling, Thomas G Beach, Geidy E Serrano, Nikhil J Pandya, Ellen Casavant, Casper C Hoogenraad, Edmond Teng, Cecilia Monteiro, Veronica Anania, Alessandro Ori, Felix L Yeh

**Affiliations:** ^1^ Genentech, Inc., South San Francisco, CA, USA; ^2^ Roche Diagnostics GmbH, Penzberg, Germany; ^3^ Banner Sun Health Research Institute, Sun City, AZ, USA

## Abstract

**Background:**

Neurofibrillary tangles (NFTs) are pathological hallmarks of Alzheimer's Disease (AD), consisting of aggregated tau protein. Tau is frequently used as a biomarker in AD clinical trials, as it provides diagnostic and prognostic information and serves as a pharmacodynamic marker to assess effects of tau‐targeting treatments. Tau PET tracers specifically bind to NFTs, allowing for tau pathology imaging. However, PET imaging requires specialized infrastructure and is costly, limiting its widespread use in trials. Our study assessed the relationship between cerebrospinal fluid (CSF) soluble tau species and tau PET imaging. The aim was to identify fluid biomarkers that could serve as more accessible alternatives for assessing tau pathology in clinical trials.

**Methods:**

CSF tau species were measured at baseline in a subset of 53 prodromal‐to‐moderate AD participants enrolled in two anti‐Tau Ph II trials (*n* = 26, *n* = 27). Tau peptides were measured by data‐independent acquisition mass spectrometry (DIA‐MS). Total tau, pTau181, and pTau205 were measured using Elecsys immunoassays. N‐term and Mid‐domain tau peptides were measured by targeted LC‐MS. pTau217 was measured using a Simoa assay and later with an Elecsys assay. Trial participants also completed [18F]GTP1 imaging. Standardized uptake value ratios (SUVR) were reported from the whole cortical gray matter and meta temporal regions using inferior cerebellar gray matter as the reference area.

46 pathology confirmed AD brains from the Arizona Study of Aging and Neurodegenerative Disorders at Banner Sun Health Research Institute were used to determine correlations between tau peptides and NFT burden in the fusiform gyrus. Tau peptides were measured by DIA‐MS; NFT burden was measured by quantifying AT8‐positive gray matter areas of adjacent brain sections. CSF and brain Tau species were correlated to [18F]GTP1 SUVR and %AT8‐positive area, respectively.

**Results:**

The analysis demonstrated CSF pTau205, pTau217, and a proteomic peptide from the 2N4R isoform MTBR region had highest correlations with [18F]GTP1 SUVR across both target regions. The same MTBR peptide was highly correlated with the %AT8 positive area in the fusiform gyrus.

**Conclusion:**

A proteomic peptide from the MTBR region was identified to be the highest correlated tau peptide in AD CSF and in the brain with tau pathology.